# Predicting motor behavior: an efficient EEG signal processing pipeline to detect brain states with potential therapeutic relevance for VR-based neurorehabilitation

**DOI:** 10.1007/s10055-021-00538-x

**Published:** 2021-09-23

**Authors:** Eric J. McDermott, Johanna Metsomaa, Paolo Belardinelli, Moritz Grosse-Wentrup, Ulf Ziemann, Christoph Zrenner

**Affiliations:** 1 Department of Neurology and Stroke, and Hertie Institute for Clinical Brain Research, Eberhard Karls University Tübingen, Hoppe-Seyler-Straße 3, 72076 Tübingen, Germany; 2 Hertie Institute for Clinical Brain Research, Eberhard Karls University, Tübingen, Germany; 3 International Max Planck Research School, and Graduate Training Center of Neuroscience, Tübingen, Germany; 4 Faculty of Computer Science, Research Platform Data Science and Vienna Cognitive Science Hub, University of Vienna, Vienna, Austria; 5 CIMeC, Center for Mind/Brain Sciences, University of Trento, Trento, Italy

**Keywords:** EEG, Brain-state decoding, Machine learning, Classification, Brain–computer interface (BCI), Motor intention, Motor behavior, Hand selection, Virtual reality, Neurorehabilitation, EEG-VR, Pre-movement, Human-in-the-loop, Open source pipeline

## Abstract

Virtual reality (VR)-based motor therapy is an emerging approach in neurorehabilitation. The combination of VR with electroencephalography (EEG) presents further opportunities to improve therapeutic efficacy by personalizing the paradigm. Specifically, the idea is to synchronize the choice and timing of stimuli in the perceived virtual world with fluctuating brain states relevant to motor behavior. Here, we present an open source EEG single-trial based classification pipeline that is designed to identify ongoing brain states predictive of the planning and execution of movements. 9 healthy volunteers each performed 1080 trials of a repetitive reaching task with an implicit two-alternative forced choice, i.e., use of the right or left hand, in response to the appearance of a visual target. The performance of the EEG decoding pipeline was assessed with respect to classification accuracy of right vs. left arm use, based on the EEG signal at the time of the stimulus. Different features, feature extraction methods, and classifiers were compared at different time windows; the number and location of informative EEG channels and the number of calibration trials needed were also quantified, as well as any benefits from individual-level optimization of pipeline parameters. This resulted in a set of recommended parameters that achieved an average 83.3% correct prediction on never-before-seen testing data, and a state-of-the-art 77.1% in a real-time simulation. Neurophysiological plausibility of the resulting classifiers was assessed by time–frequency and event-related potential analyses, as well as by Independent Component Analysis topographies and cortical source localization. We expect that this pipeline will facilitate the identification of relevant brain states as prospective therapeutic targets in closed-loop EEG-VR motor neurorehabilitation.

## Introduction

### Motivation

This study is part of a research project that aims to develop a personalized “closed-loop” therapeutic VR environment for patients with stroke, where the parameters and the timing of visual stimuli in the virtual world are determined by the ongoing EEG such as to optimize the efficacy of VR-based neurorehabilitation. The approach is based on the hypothesis that slow fluctuating brain states modulate motor behavior (Schmidt et al. 2016) and that synchronization of suitable target brain states with the timing of visual stimuli in the VR world (i.e., signals to initiate paretic limb movement) can optimize therapeutic efficacy of VR-based physiotherapy. Results from real-time EEG-triggered transcranial magnetic stimulation (TMS) support this notion, where the exact same stimulus has been shown to have different effects depending on EEG-defined oscillatory brain states, both in an instantaneous sense, and with regard to the induction of long-term plastic changes (Zrenner et al. 2018). Here, we use pre-movement EEG data from 9 healthy volunteers performing a simple repetitive reaching task to develop an open-source EEG-signal processing and classification pipeline. Our pipeline is designed to identify ongoing brain states predictive of planning and execution of movements as potential therapeutic targets in motor neurorehabilitation, while also taking into account the practical limitations of implementing real-time EEG-classification in a clinical setting.

### Neurophysiological background

Various characteristic EEG signals have been described in relation to the preparation and execution of movements: a low-frequency negative shift in the EEG recording, termed the movement-related cortical potential (MRCP), occurs about 2 s before voluntary movement (Shibasaki et al. 1980). The MRCP is an encompassing term which can be separated into the *Bereitschaftspotential* (BP) (Kornhuber and Deecke 1965) in self-paced voluntary movement tasks, and as *Contingent Negative Variation (CNV)* (Walter et al. 1964) in cued movements, concerning the movement preparation period between a *warning cue* and a *go cue*. The BP itself consists of two components: the early BP, characterized by a negative potential with a maximum over the centro-medial cortex, and the late BP (also known as the *Lateralized Readiness Potential,* LRP) occurring 400 ms prior to movement onset, lateralized to the contralateral hemisphere around EEG electrodes C1 or C2 (Shibasaki and Hallett 2006). CNV also consists of two distinct waves (Rohrbaugh and Gaillard 1983): the fronto-centrally dominant wave and the centro-parietally dominant wave. The former tends to reflect orienting properties of the warning signal (Loveless and Sanford 1974), while the latter is thought to reflect motor preparation, and to be mostly identical to the readiness potential (Rohrbaugh and Gaillard 1983), although it contains some non-motoric components and is more strongly influenced by the amount of pre-cue information (Brunia 2003; Ulrich et al. 1998). A further neural correlate of movement is the event-related desynchronization (ERD) occurring in the mu and the beta frequency bands (Pfurtscheller 1981; Pfurtscheller and Aranibar 1979). In contrast to MRCP components, ERD onset in the beta band is bilateral in primary motor areas during movements of the non-dominant side, and contralateral when performing movements with the dominant side (Bai et al. 2005). All of these components are indicative of motor planning and upcoming motor behavior (Shakeel et al. 2015).

The period prior to movement can also be defined as “pre-movement,” and is referred to as the planning or preparation phase of movement (Crammond and Kalaska 2000; Toni and Passingham 2003). Typically, this period is thought to represent the time in which participants are conscious that they *will* make an action, but it is before any muscle activity can be registered. Libet et al. (1990, 1983) showed that unconscious motor preparation precedes conscious initiation of the action, at least according to the current definitions of motor-related brain activity. Therefore, the aforementioned MRCP components could be thought of as a neurological marker predicting *whether* and *when* a spontaneous movement will occur (Schultze-Kraft et al. 2017), according to the “*What, When, Whether Model of Intentional Action*” (Brass and Haggard 2008). whereas the later MRCP components, as seen in studies by Coles (1989) and Eimer (1998), provide strong evidence as a neurological marker for predicting *what* kind of movement will occur (i.e., left vs right).

### Task design

For the purpose of developing a suitable signal processing and classification pipeline, we use an implicit repetitive two-alternative forced choice task (use of the right or the left hand in a simple reaching task) in order to identify brain states that are relevant to “*what”* motor behavior is being planned, and using single-trial classification accuracy as the measure of pipeline performance. This task is neither self-paced (as it would need to be to express the classical BP, where participants move at their own volition without external cuing), nor does it include “get ready” cues leading to an explicit movement preparation stage or instructions as to which hand should be moved (as used in tasks designed to express the CNV and LRP). Indeed, in our study, the delay to the appearance of the visual stimulus is randomly jittered in order to reduce predictability and to investigate whether the natural ongoing brain state at the time of the stimulus biases the motor choice that is then freely made. This design enables us to investigate ongoing brain dynamics that are expressed *before* the start of motor preparation, and which may nevertheless bias an upcoming motor behavior. A further advantage of this task is that it is similar to the physiological situation of reaching during motor rehabilitation training. However, without an explicit preparation period, the neurophysiological interpretability of our task is more difficult as compared to well-established classic paradigms (see above). With regard to decoding motor choice from EEG, previous studies have typically yielded classification accuracies ranging from 60–82.5%, depending on movement complexity and the classifier algorithms (Bai et al. 2007; Doud et al. 2011; Haw et al. 2006; Jiang et al. 2015; Jochumsen et al. 2013; Lew et al. 2012; Liao et al. 2014; Niazi et al. 2011; Tavakolan et al. 2017; Vuckovic and Sepulveda 2008; Waldert et al. 2008; Yong and Menon 2015).

### EEG-based classification

Many different EEG-classification approaches exist, mostly in the context of Brain-Computer Interface (BCI) applications. These can generally be divided into the following three processing stages, each with its own set of different design choices:

1. *Features of Interest:* What aspects of the EEG signal are relevant and during what time window? With regard to motor preparation, researchers have used low-frequency oscillations (Pereira et al. 2017), event-related potentials (ERP) between channel-pairs (Schultze-Kraft et al. 2017), independent component analysis (ICA) (Karimi et al. 2017) and event-related (de)synchronization (ERD/ERS) of oscillatory power at 10 and 20 Hz (Morash et al. 2008; Müller-Putz et al. 2008; Pfurtscheller et al. 2006; Waldert et al. 2008; Yong and Menon 2015); all have been found to be informative.
2. *Feature Extraction:* It is then necessary to reduce the number of dimensions and extract those that are most relevant, while excluding noise. Blind source separation methods such as ICA (Karimi et al. 2017) or principal component analysis (PCA) (Velu and de Sa 2013) are frequently used, as is the extraction of power (and phase) at a given set of frequencies from a time-series using the discrete Fourier transform (Planelles et al. 2014).
3. *Classification Algorithms:* How can different classes best be differentiated? Various methods beyond linear regression have been developed such as Support Vector Machines (Liao et al. 2014; Tavakolan et al. 2017), Linear Discriminant Analysis (Bhattacharyya et al. 2010), Neural Networks (Atzori et al. 2016; Loukas and Brown 2004; Roy et al. 2012), Boltzmann and Deep Belief Networks (Chu et al. 2018), *K*-nearest neighbors (Blankertz et al. 2002), or even custom algorithms (Bai et al. 2011).

Another relevant parameter in the case of EEG concerns the number and location of EEG electrodes, which spans from 4 to 256 in the literature (Bai et al. 2011; Hammon et al. 2007; Meinel et al. 2016; Pfurtscheller et al. 2006; Planelles et al. 2014).

In summary, there are a large number of free parameters in how a given pipeline can be setup, and different researchers make different choices (with consequences for reproducibility, see (Botvinik-Nezer et al. 2020)). Given the many degrees of freedom, we here investigate how the most relevant parameters influence classification performance in the specific case of decoding motor choice from EEG at the time a visual target for a reaching task appears, and determine suitable default settings. Further background on machine learning, algorithms, and feature selection/extraction can be found in Appendix 2.

### Need and novelty

This study is intended to help realize the full potential of the individual EEG signal to improve the effectiveness of VR-based therapy by personalizing the VR task according to brain activity. Here, we present and make available a novel signal processing pipeline able to differentiate EEG-defined brain states predictive of movement intention, which we hope may serve as therapeutic targets in future “closed-loop” EEG-VR therapy paradigms. To facilitate use of such an approach in a clinical setting, we have systematically investigated and identified suitable *default values* for relevant parameters in the analysis pipeline (number and location of EEG channels, time windows for analysis, feature extraction method, classification algorithm, and minimum number of trials for effective training in a calibration phase), that lead to a personalized and individualized classification model that can then be implemented in a real-time “closed-loop” EEG-VR paradigm.

A further point of novelty with respect to previous studies is that we have chosen a simple reaching task that mimics a therapeutic setting, as to make the results more directly applicable. One application of an EEG-VR therapy system with the ability to detect real-time EEG-derived brain states corresponding to a patient’s motor intention is the perception of *virtual movements* of the affected limb in the VR-world that are appropriately synchronized to the patient’s endogenous movement intention. This could be helpful in cases where the patient has severe motor impairment, such as in hemiplegia, where no voluntary movements can be generated in the affected limb. The approach is similar to mirror therapy (Dohle et al. 2009; Yavuzer et al. 2008), where the illusion of a successful movement of the affected limb supports the rehabilitation process, but in our case the illusory movement is instead coupled to and driven by the stroke-affected networks instead of the unaffected hemisphere. We believe that there is significant potential to further develop such paradigms using EEG and VR to improve rehabilitation outcome in patients.

## Material and methods

### Participants

The study protocol was approved by the local Ethics Review Committee of the Medical Faculty of Eberhard Karls University Tübingen (Protocol 716/2014BO2). The study was conducted in accordance with the latest version of the Declaration of Helsinki. After giving written informed consent, 11 right-handed healthy male participants (mean and standard deviation age 27 ± 5 years, range 22–40 years) were included in the study fulfilling the following pre-established inclusion criteria: (i) age 18–65 years, (ii) no known medical conditions, (iii) self-identified as right-handed. Two participants were excluded, and their data were not further analyzed, one due to a strong bias toward use of the right hand (> 95% of trials), such that fewer than 50 trials remained in both classes, which is insufficient to train and test a classifier; another participant was excluded due to EEG artifacts affecting > 50% of the trials. The number of included participants is similar to previous studies in this domain (Wierzgała et al. 2018).

### Experimental setup

Scalp EEG was recorded using 126-channel high-density EEG cap (Easycap GmbH, Germany) in a standard 10–5 system layout (Oostenveld and Praamstra 2001), and right and left deltoid muscle activity was recorded using bipolar surface EMG electrodes (Kendall, USA) placed on the belly of the anterior deltoid and referenced to the acromion. EEG and EMG signals were acquired simultaneously using a biosignal amplifier (NeurOne Tesla, Bittium, Finland) at a sampling rate of 5 kHz in direct current (DC) mode while applying an anti-aliasing low-pass filter with a cutoff at 1,250 Hz and no high-pass filter. Participants were seated in a comfortable reclining chair, while visual stimulation was provided using an immersive curved display (CF791, Samsung, curvature 1500R, 34″ diagonal) at a distance of 1.5 m to achieve equidistance. A custom-built photodiode was attached to the screen to record the timing of visual stimulus onset on an additional bipolar input channel of the biosignal amplifier. An infrared hand tracker was used (*Leap Motion Orion v4,* Ultra Leap, USA) to monitor hand position using a refresh rate of 100 Hz. Additional hand tracking markers to record movement onsets were taken from the Leap Motion and measured through Unity3D software (Unity Technologies, USA). These were calibrated to correspond to the participants’ hands relative to a *home position*, in which the participants’ began each task. Timestamps were recorded for every stimulus presentation, hand movement into and away from the home position, and stimulus contact. Synchronization was achieved between the photodiode and Unity3D data through timestamp alignment.

### Virtual reality presentation

The visual stimuli presentation and task logic was programmed in C# and realized through Unity3D, it is an adapted form of a protocol used in a previous study (McDermott and Himmelbach 2019). Stimuli consisted of yellow squares (2.5 cm × 2.5 cm, visual angle ~ 1.0º) on the immersive display, centered along the midpoint of the participants’ body position. The position of the participants’ hands as tracked were also presented virtually on the screen. The stimuli would stay present until the participant contacted them with their virtual hand. These stimuli were precisely tracked in time using two methods: (i) internal timestamps via Unity3D, and (ii) a photodiode which was connected directly into the EEG-NeurOne interface that placed triggers in the EEG-trace upon stimulus presentation.

### Study design

**Fig. 1:**
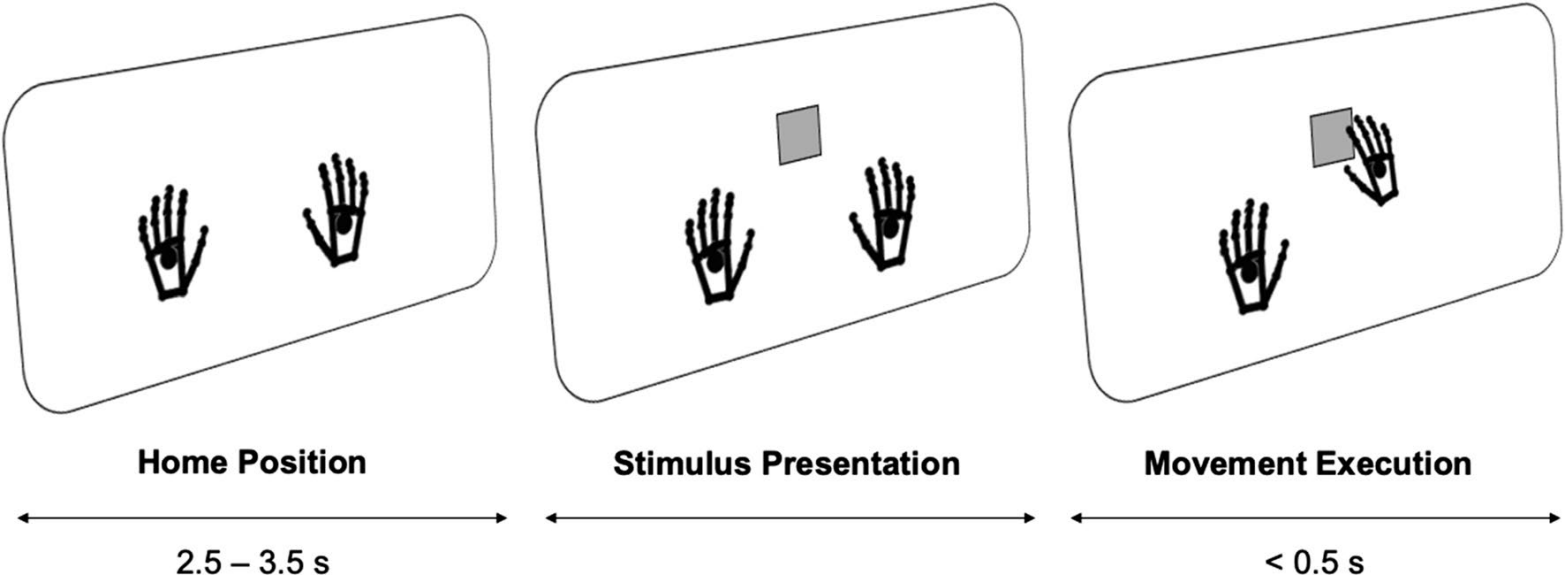
Study and task design. The participants started with their hands in a calibrated home position. After a random interval, a visual target stimulus was presented along the central vertical of the screen. The participants then reached out with either the right or the left hand and contacted the virtual stimulus, which then disappeared and the participants returned their hands to the home position. EEG was recorded during the task

Following a 5-min eyes-open resting-state EEG measurement, participants were instructed to place their hands on marked spots (*home position*) on a glass board and to remain at rest. Upon presentation of a target stimulus, the participant was then instructed to reach out to contact the stimulus in virtual space, which disappeared on virtual contact, and then to return to the home position and wait for the next stimulus to appear. After reaching the home position, the next stimulus appeared after a jittered interval of 2.5–3.5 s; this timer was reset if the hands moved from the home position prematurely. Target stimuli were presented in the center of the screen, with an added ± 0.25 cm random jitter along the x-axis, and over three stacked positions on the y-axis (as if the squares were stacked on top of one another). After a short demonstration consisting of 10 stimuli, the participant began the task. Participants completed a total of 5 rounds consisting of 216 stimuli with short breaks between rounds, yielding 1080 trials in total. After this, another 5-min eyes-open resting-state EEG measurement was performed. Note that participants were not instructed in any way with regard to hand selection and were free to select either their right or left hand when making the reaching movement. This means it was theoretically possible for a participant to use the same hand the entire time; we accepted this drawback in order to avoid biasing the participants, and we discuss this aspect of the design (hand-use preference bias) extensively below (Fig. 1).

### Data Preprocessing

#### Software, filtering and epoching

Data processing was performed with custom scripts in MATLAB (R2017b, MathWorks Inc.), using EEGLab Toolbox v. 13_6_5b (Delorme and Makeig 2004), FieldTrip toolbox v. 20,190,705 (Oostenveld et al. 2011), and FastICA toolbox v. 2.5 (Hyvarinen 1999). After confirming alignment of the timestamps from Unity3D and the photodiode, EEG and EMG channels were saved separately. Then, a high-pass filter was applied to the EEG raw data (sample rate 5 kHz, 1 Hz cutoff, FIR filter order 5000). EEG Epochs were extracted centered ± 2.5 s around the onset time of the visual stimulus, downsampled from 5 to 1 kHz (applying a zero-phase anti-aliasing filter), and low-pass filtered (45 Hz cutoff, FIR filter order 500). Epochs were then split into a pre- and post-stimulus segment, and the further data cleaning steps were performed based on the signal properties of the pre-stimulus segment only. Post-stimulus data were not used to inform the cleaning process to ensure that there was no way for the EEG signals or any artifacts during the task execution period to indirectly affect the pre-stimulus signal through the cleaning process.

#### Bad channel and trial rejection

Bad channels and trials were manually rejected using FieldTrip’s “ft_rejectvisual” function by visually checking for outliers according to variance. Peripheral electrodes that were affected by the reaching movement were removed by default; the list of removed peripheral electrodes can be found in Appendix 2.

#### Artifact reduction

Data were then re-referenced to the average signal across the remaining channels and baseline-corrected. Next, ICA was performed using the FastICA algorithm on each participant independently, setting the number of components to 50 and using the symmetric mode of the algorithm with a Gaussian contrast function. Components were visually inspected with respect to topography, time-course, and the resulting channel-level signal amplitude. Specifically, ocular artifacts were removed, and channels AFp1 and AFp2 were checked for confirmation of the removal of eye-blinks and eye-movements; on average 2 components were removed. The same transform and ICA component removal was applied to the post-stimulus to re-create a single cleaned dataset containing the entire − 2.5 s to 2.5 s window.

#### Detection of movement onset

**Fig. 2:**
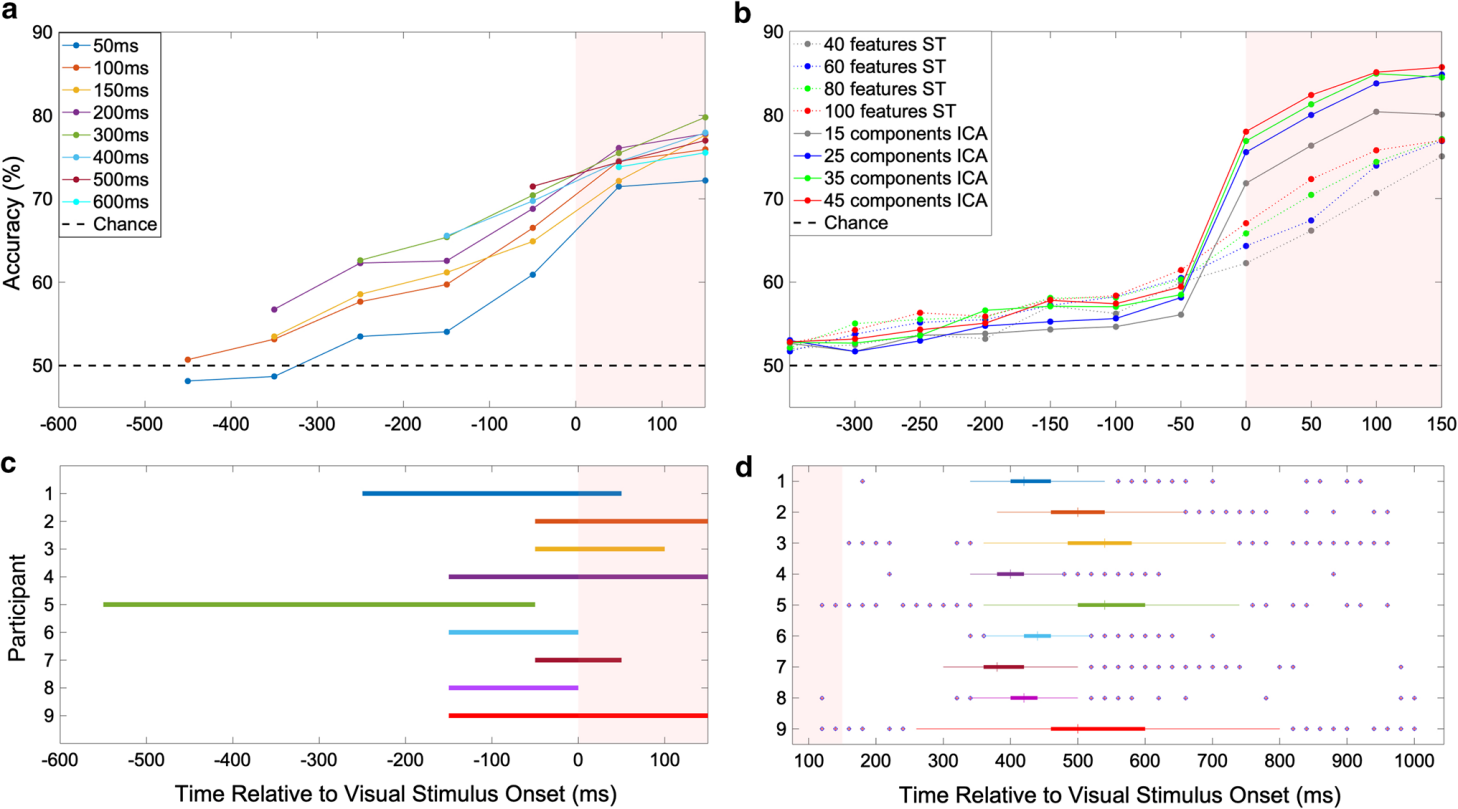
Time window analysis and movement onsets. **a** Function of classification accuracy with respect to data window position and length. Prediction accuracy is averaged over the group of 9 participants, the position of the data point on the x-axis shows the right-side end of the time window. **b** Group average classification accuracy for different feature extraction methods (ICA vs. PCA-based spatiotemporal) at different time points, with varying number of features, using a fixed time window 150 ms prior to and including the data point shown. **c** Optimal time windows for each participant. The horizontal bars represent time windows which produced the highest accuracy in the classification, with all other variables held constant. **d** Overview of movement onsets across participants, relative to the visual onset of the stimulus, as determined by hand position tracking. The median latency and interquartile range is shown with whiskers extending to the most extreme data points excluding outliers, plotted separately. The shaded pink area in all figures represents the time from the onset of the visual stimulus to 150 ms after the stimulus, which is data still used by the classifier. 21 trials are contained in this area (0.02%)

Movement onset times were determined using the hand tracking device, defined as the time when the hands moved away from the home position, and these times were correlated with EMG data to ensure accuracy. The EMG data were filtered and an envelope was created (Appendix 1; Fig. 9), the movement onset according to EMG was defined as the first time that the EMG envelope exceeded 50% of its maximum. The movement onset distribution is visualized in Fig. 2d. We allowed classification using time windows up until 150 ms *after* visual onset in many of our parameter optimizations, as no muscle activity should be present at that time according to prior research (Ladd and Woodworth, 1911; Murakami 2010). However, in our data, 21 out of 9720 trials (0.02%) showed signs of muscle activity in this period.

#### Classifier data preparation

Each data trial was labeled according to the hand selection (i.e., left vs right). In order to achieve the most robust output from our classifier, for each participant, the trial order was randomly permuted and then split into two sets: first, training data (80% of a participant’s data) and second, testing data (20% of a participant’s data). The training data were used for model building and cross-validation. The testing data were kept separate and used to assess the final model for accuracy, where accuracy was reported as the percentage of correctly classified trials.

The classifier was trained with an equal number of examples for right vs. left arm movement to avoid a priori bias. However, because participants chose their arm freely in the reaching task (see below), the maximum number of available training samples is limited by how often the rarer side was chosen. Due to varying inter-individual biases, the maximum number of trials available varied between participants: After balancing, the datasets ranged from 100 to 296 trials in each class, with a mean of 225 trials.

#### Hand-use preference bias and dataset size

Given that the paradigm involved a free choice of using the left or the right hand, we calculated the preference of using the right or the left hand on a trial-by-trial basis and searched for repeating patterns. We created a 2 × 2 movement matrix (Appendix 1 Fig. 11), in which we predicted the likelihood that a right-hand trial would follow a right-hand trial, as well as the likelihood that a left-hand trial would follow a right-hand trial. This was also calculated for the opposite cases. As seen in the section above, these individual-level biases caused participant-level fluctuations in the number of available trials, as we performed *post hoc* balancing between left- and right-hand trials in order to set the a priori chance of classification to 50% for each condition.

### Classifier properties

The classifiers included linear support vector machines (SVM) and logistic regression with dimensionality reduction and features derived from principal component analysis (PCA) and independent component analysis (ICA). All of the methodology, described in detail in the following and further in Appendix 2, was implemented using MATLAB, FastICA, and the FieldTrip toolbox, along with custom code.

#### Feature extraction with spatiotemporal PCA

To extract spatiotemporal features in a given time window for decoding, we concatenated the respective data matrix within the chosen window in each trial of the training data into one long spatiotemporal representation vector. In the simplest case, when the time window constitutes only one time instant, the vector length equals the number of channels. The entries of this vector could directly be used as input features for the classifier, as done in Ofner et al. (2017). However, a wider time window leads to a channel × time spatiotemporal matrix with a large number of non-independent elements. Therefore, to reduce data dimensionality, PCA was applied to the spatiotemporal matrices, which were then projected into the subspace spanned by *M* eigenvectors corresponding to the *M* largest eigenvalues to get the compressed data. The entries of the compressed spatiotemporal vectors were used directly as features for classification.

#### Feature extraction with ICA

After a dimensionality reduction step, and before running ICA, we subtracted the average data epoch over all trials from each individual trial. This preprocessing step has been shown to eliminate bias in the ICA results due to temporally overlapping brain activity that is phase-locked to the stimulus onset (Metsomaa et al. 2014). ICA was then applied to this pre-processed data using FastICA with symmetric mode and the *“tanh”* contrast function to estimate *M* independent components. An ICA-derived demixing matrix was used to separate the components from the compressed data, where the mean and the variance of each component was selected as features to be used in single-trial-level classification. Here, the variance of a component was computed after subtracting the averaged component over the training trials from the single-trial waveform, where the mean of this mean-subtracted component was assumed to be zero. Therefore, in the ICA approach, where *M* is equal to 30, the total number of features would equal 60.

#### Classifier selection

We used the MATLAB *fitclinear* function with fivefold cross-validation and the parameters as summarized in Table 1 to build linear classifiers for predicting the choice of hand based on the EEG-derived features described above. Here, we use the notation (“*option name*,” “*option choice*”) to describe the choices we made when using the fitclinear function. Logistic regression (“*Learner,” “logistic”*) was chosen for the linear classification model (after testing against SVM). Regularization was performed with the lasso (L1-norm) penalty (“*Regularization,” “lasso”)* for the ICA-derived features, and with the ridge (L2-norm) penalty for the spatiotemporal PCA-derived features. Sparse Reconstruction by Separable Approximation (SpaRSA; “*Solver,” “sparsa”)* was the objective function minimization technique for ICA, and a stochastic method was used for the spatiotemporal PCA. Lastly, automatic optimization of the regularization coefficient was performed via *('OptimizeHyperparameters', 'Lambda').* This optimization attempts to minimize the cross-validation loss (error); specifically, it searches for this optimum among 30 positive values of the regularization coefficient, log-scaled in the range [10^−5^ / number of trials, 10^5^ / number of trials]. The above-mentioned parameters and options were chosen after preliminary test runs comparing parameters options.

**Table 1:** Feature extraction approaches

Approach	Classifier	Linear model	Regularization penalty	Minimization technique	Lambda
ICA	MATLAB *fitclinear* fivefold cross-validation	Logistic	Lasso (L1)	SpaRSA	Optimized in-function (30 times per iteration)
ST	MATLAB *fitclinear* fivefold cross-validation	Logistic	Ridge (L2)	Stochastic Gradient Descent	Optimized in-function (30 times per iteration)

### Parameter optimization

We tested our data separately using *fitclinear* with spatiotemporal PCA- and ICA-derived features, as well as across different time windows, various sets of channels, and differing number of trials.

#### Determining optimal time windows

To find the optimal time window for each participant, we tested classification accuracy for different window lengths and window positions. The position of a window is here defined by its start time with respect to the onset of the visual stimulus. To find the optimal window size, its position was first set at − 550 ms, and the size then varied, with possible sizes being 600, 500, 400, 300, 200, 150, 100, and 50 ms. Simultaneously, to find the optimal position of the time window, we shifted each aforementioned window 100 ms at a time until they reached the extent of our classification window (at 150 ms). In this format, the window size of 600 ms would have only 2 plausible positions, (− 550 to 50 ms, and − 450 to 150 ms), whereas the window size of 100 ms would have 14 test positions. For each window size and position, the classification accuracy was evaluated by averaging the results over 20 randomly generated and non-overlapping training and test sets.

#### Determining optimal channels

The question of how many channels are needed to create an effective pipeline is of practical relevance especially for clinical applications. To determine the most informative set of channels for classification, we considered subsets of each participant’s channels *post hoc* using an iterative process: Starting with a single channel and a time window of −150 ms to 150 ms, ICA feature extraction was performed, and then the classification accuracy of each single channel was compared, with the most informative channel being selected. Then, the process was repeated and the classification accuracy of n + 1 channels (the previously selected channel(s), with each remaining channel, separately) was compared, choosing again the channel with the highest accuracy. This was repeated until n = 5, at which point the classification accuracy of n + 3 channels was compared until n = 30, at which point the n + 5 channels were compared until all channels were selected. For this analysis, the *training data* and the calculated optimal time windows were used in a fivefold cross-validation manner, the resulting accuracy was assessed using the *validation data*.

Each channel was given “points” based on its ranking: channel rankings #1–5 = 5 points, #6–15 = 3 points, #16–30 = 2 points, #31–50 = 1 point, > #50 = 0 points. Through the addition of these points, we compiled a list of channels for each participant, where the most points would indicate the most relevant channel for classification. Then, we took these participant-specific lists and combined the point values to create the optimal channel set at group level. If an individual participant was missing a channel, and therefore the group list had, i.e., only 8 of 9 participants contributing point values to the specific channel, the missing points were replaced by the average of the other participants. The group-level ranking points were normalized to 0–100 with respect to the single channel with the highest score.

#### Classifier features

We determined the number of features that should be used by testing the classifier performance using the ICA approach with 5, 15, 25, 35, and 45 components, and the spatiotemporal approach comparably with 40, 60, 80, and 100 features. Other variables were held constant, *i.e.*, all available channels were used, and a sliding 150 ms time window was used across various starting points.

#### Number of trials

To estimate how classification accuracy improves with an increasing number of trials, we ran the pipeline with our recommended parameters taken from the results of the previous analysis. The training set size was increased in a controlled manner, with trials being equalized for left- and right-hand choices. We started with 10 randomly selected trials for each case, and increased the number by 20 until reaching the maximum number of trials, which was determined by the minimum of total left- or right-hand selections (leaving out 20 test trials in both selection categories). These training data were used to build a classifier, which was tested against the 40 test trials. This process of shuffling the training and the test datasets was repeated 20 times.

## Real-time analysis

The goal of this study was the development of a processing pipeline that can be implemented in real-time EEG-VR paradigms. Whereas the calibration of the classifier based on training data is time demanding, this step is only required once and does not need to run in real time. All of the computations of the actual trial-by-trial processing and classification using a sliding window of data can be performed in real time by standard PC hardware. In an actual real-time implementation, an EEG amplifier is required that provides a continuous low-latency low-jitter stream of real-time data (3–5 ms delays are possible with standard commercial hardware). Additionally, care needs to be taken to minimize and compensate for signal delays due to digital signal processing steps such as digital filters, see previous work on a real-time EEG-based oscillatory phase estimation method implemented using Mathworks Simulink Real-Time (Zrenner et al. 2018) as an example for such an implementation. In our case, a linear transformation of the data acquired in the sliding window is performed using the ICA weights from our calibration. These data are then fed into the participant’s saved model and a movement class is predicted.

In the context of this study, we performed two real-time simulations: (1) using a time window prior to movement onset (here, *the type of the stimulus* in the VR world can be controlled, i.e., *virtual movements*), and (2) using a time window prior to visual stimulus onset (here, *the type and position of the stimulus* can be controlled by EEG, i.e., *stimuli positioning*). Therapeutic applications exist for both use-cases. To perform these simulations, we used each participant’s first 100 right-hand and first 100 left-hand trials to calibrate a classifier using our recommended parameters. To test the model, the simulation then classified all remaining trials, starting from one trial after the last trial indexed calibration. This was done without order permutations or correction for bias (to include any non-stationarities that would also be present in a non-simulated real-time environment). Simulations were only performed for study participants with at least 25 testable trials after the calibration phase; thereby excluding participants 3 and 9.

## Neurophysiological analysis

### Interpreting the classifier weights and the ICA topographies

For this subanalysis, our aim was to study whether the estimated classifier and independent components could be used to gain insight into the relevant neurophysiological processes behind movement planning and preparation. Specifically, we addressed the question “*From where are the relevant signals arising on the cortex?*”. The topographies of the relevant EEG signals, as determined by the classifier, can then be used to estimate the cortical source activity. They can also be compared across participants with the goal of possibly using the similarities over participants as a priori information to increase the accuracy of classification with reduced number of trials in the future.

The topography ν reflects the spatial distribution of the brain signal whose mean over the chosen time window is most relevant for classification. In our case, ν represents the topography which would be most predictive of right- versus left-hand movement. In Appendix 2, we provide a detailed derivation of this relation.

### Time–frequency analysis

Time–frequency analysis was performed using Morlet wavelets with a width of 7 cycles over the C1 and C2-centralized areas for each respective hemisphere (electrodes FCC1h, C1, CCP1h, FCC3h and their counterparts). In this analysis, frequencies from 2 to 40 Hz were analyzed over a time window of −150 to 150 ms relative to the visual stimulus. This transformation was applied for the right-hand trials and then the left-hand trials, and a difference was calculated through the operation (X_1_ − X_2_)/(X_1_ + X_2_), where X_1_ is the output from the right-hand trials, and X_2_ is the output from the left-hand trials time–frequency analysis.

### Source Localization

To more accurately place our most relevant classifier features (i.e., the topography reflected by ν) within a neurophysiological framework, we localized cortical sources of relevant activity using Minimum Norm Estimation (MNE) (Hämäläinen and Ilmoniemi 1994) on a brain cortical mesh, with a regularization factor of 10^–16^, and free-oriented dipoles in the forward model.

In this study, we did not obtain individual MRIs for each participant, but we did use the 10–05 EEG fitting standards; this allowed us to use 10–05 electrode positions and the standard *Boundary Element Method* head model provided by Fieldtrip (Appendix 2). Along with this, we used a standard source-model based on the description of a surface within the grey matter of the Buckner40 brain (Fischl 2012). This procedure resulted in a cortical sheet with 15,684 unique source points. After specifying a participant’s unique remaining electrodes, these models were plotted together to ensure fit.

## Results

### Summary

Although we do analyze the neurophysiological plausibility of the resulting optimized features with regard to predicting lateralized movement, the main focus of this study is the development of a clinically applicable EEG signal processing and classification pipeline. With respect to this, we first present the results of optimizing the (1) feature selection and classification method, (2) temporal selection of relevant time window, (3) spatial selection of EEG channels, and (4) number of trials; followed by the neurophysiological results.

### Feature and classifier selection

In order to investigate the effect of the number and type of features on the prediction accuracy, a 150-ms sliding window was chosen in combination with several different feature conditions. Within the ICA approach, two main learners were tested: SVM and logistic regression. We thoroughly tested each classifier over 50 iterations for each participant, and the results showed that logistic regression returned slightly higher accuracies across 6 of 9 participants when compared to SVM. Given this result (see Appendix 1, Fig. 10), in all subsequent analyses we used a logistic regression learner with ICA.

Additionally, ICA consistently results in higher classification accuracies than the spatiotemporal approach once the time window incorporates −150 ms onwards, regardless of the number of features, with the accuracy saturating above 25 components (Fig. 2b). Even the smallest tested feature set from ICA (15 components, i.e., 30 features) resulted in a greater accuracy than the largest feature set of the spatiotemporal approach (100 features). We therefore determined 30 ICA components to be a suitable choice.

### Determining optimal time window

For the analysis described above, a 150-ms sliding window was used. Here, we examined the optimal time window position and length by comparing different possible combinations for each study participant. Our data indicated that the highest accuracy when averaged over the group was from the time window −150 to 150 ms (Fig. 2a). The size and position of the sliding window was optimized for each participant in terms of producing the highest validated classification accuracy. Our results showed the average *optimal* window size was 239 ms, with the averaged range being from −172 to 67 ms with respect to the visual stimulus (Fig. 2c). We therefore determined a window size of −150 to 150 ms as a suitable choice for the pipeline, encompassing the optimal windows for most study participants.

## Ranking of EEG channels

**Fig. 3:**
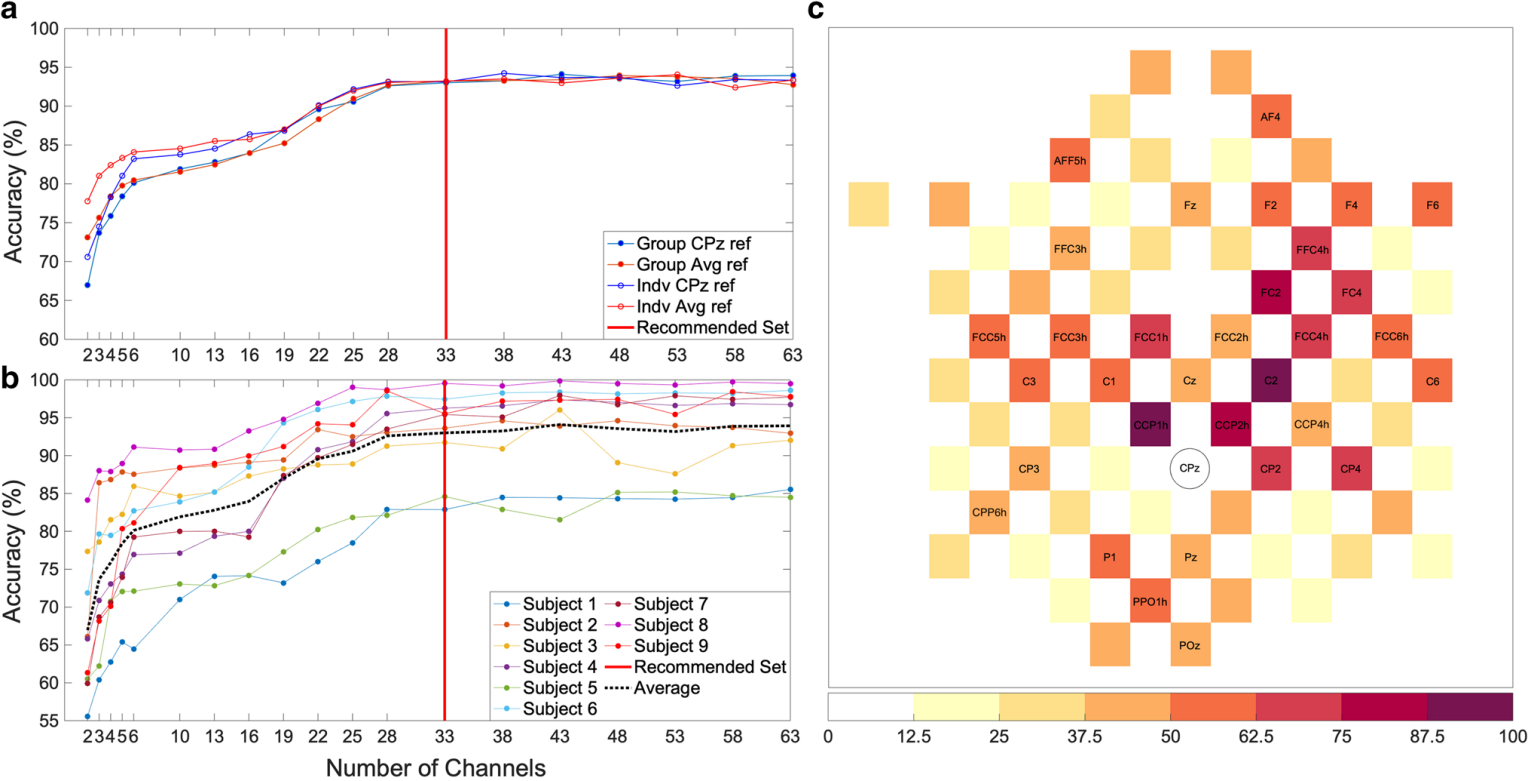
Channel selection and recommended set. **a** Group averaged classification accuracy as a function of number of channels when comparing a standard set of group optimally ranked and individually optimally ranked channels for both CPz-referenced and average-referenced EEG data. Accuracy is determined from fivefold CV. **b** Classification accuracy with additional channels at participant level using CPz-referenced data with the group-optimized ranking of electrodes. **c** Channels ranked by prediction accuracy for the group, the normalized ranking scale represents the group’s most important electrodes ranked from worst (0) to best (100). The labeled electrodes represent the 32 chosen electrodes for the pipeline, plus CPz as a reference (marked)

As shown in Fig. 3a, the average classification accuracy as determined through a fivefold cross-validation increases with the number of channels used, plateauing around 30 channels. Conditions included using optimized channels with respect to individuals and to the group as a whole, as well as comparing between average- and CPz-referenced EEG data. Granted, we chose to use the CPz-referenced dataset in our pipeline, as using average-referenced data with few channels is not realizable in practice. A minimum of 64 electrodes (Nunez and Srinivasan 2006), evenly spaced and covering over 50% of the head surface (Luck 2014), are typically required for properly computing an average reference.

According to our results, there is a small benefit of using individually optimized sets of channels when using fewer than 20 channels. Individually optimized CPz-referenced channels do show a small detriment in accuracy compared to average-referenced channels when using fewer than 10 channels, likely due to the aforementioned methodological implications of using average-referenced data. Individual data are shown in Fig. 3b and display variability between participants with regard to the achievable accuracy, but show a comparable marginal benefit of including additional EEG channels up until ~ 30 channels. Based on this result, we propose a set of 32-channels that have the highest average ranking in terms of their cumulative predictiveness as shown in Fig. 3c and listed in Appendix 2.

## Number of calibration trials

**Fig. 4:**
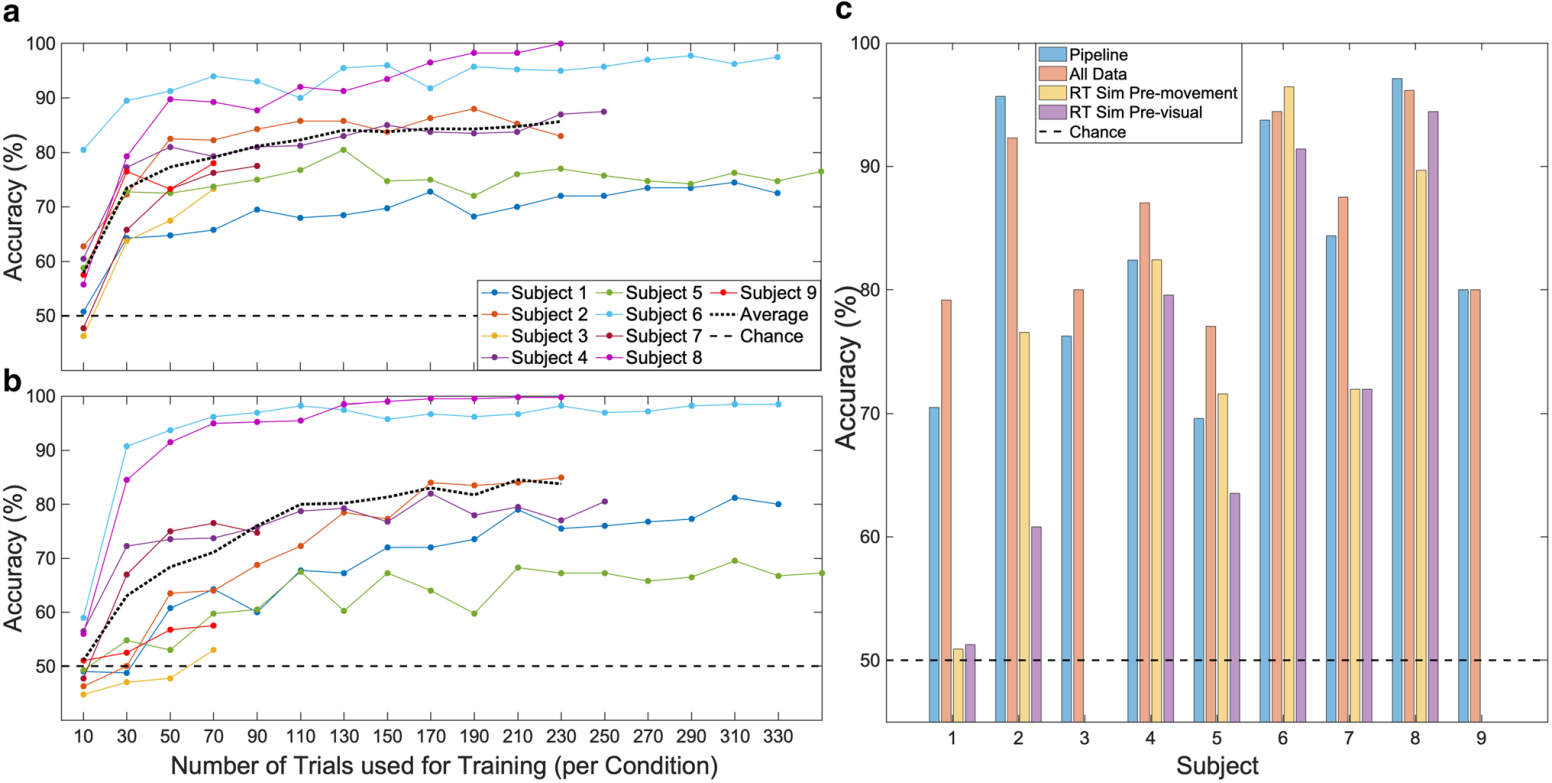
Calibration trials and final accuracies. **a** Prediction accuracy as a function of the number of trials used for classifier training, with parameters as in the recommended pipeline, using a time window of −150 to +150 ms around the onset of the visual stimulus. Training and test data were shuffled 50 times for each data point. **b** As a), but with a time window limited to before the visual stimulus (−150 to 0 ms). **c** Final accuracies for four conditions: the recommended pipeline vs all available data vs real-time simulation (pre-movement and pre-visual). All accuracies are obtained by testing against never-before-seen testing data. The blue bars represent a classifier condition with parameters recommended from our pipeline: the suggested set of 32 channels, a time window of −150 to +150 ms, and 100 trials per condition for training. The red bars represent a classifier condition in which all available trials and electrodes were used for the same time window. The yellow bars represent a condition where after a calibration step using the recommended parameters was completed (including using only the first 100 trials in each condition for training), a model was then made and trials were classified in a simulated real-time manner. The purple bars are the same as the previous, with the exception of the time window used being from −150 to 0 ms. Participant 3 and Participant 9 were excluded for both real-time simulations, as they had fewer than 25 trials after calibration was complete, whereas the remaining participants had a mean of 422 testable trials (range 171 to 573) after calibration

We calculated the number of trials required in the calibration session to achieve a given classification accuracy (see Fig. 4a) when using the set of 32 EEG channels determined above, a fixed time window from −150 to 150 ms with respect to the visual onset of the stimulus, and the ICA approach for classification with 30 components (or *M−1*, if there are less than 32 EEG channels). This analysis indicated that additional calibration data beyond 100 trials per condition brings only marginal improvement on average.

## Classification accuracy in the causal case for EEG-dependent stimulation

In therapeutic paradigms where the timing of a visual stimulus (e.g., in the VR environment) is determined by real-time classification of ongoing neural oscillations as recorded by EEG, only the data preceding a potential stimulus are available to the model. This would for example be the case in a setting where a patient is presented with a stimulus in the VR world that is positioned according to detection of the brain state corresponding to the impaired limb (i.e., they *intend* to use the impaired limb, and therefore they are presented with a challenging but achievable stimulus for their range-of-motion). In order to quantify the prediction accuracy in such a scenario, we used the same optimized parameters as above regarding trials, but with only pre-stimulus EEG data (time window −150 to 0 ms), as shown in Fig. 4b.

## Summary of recommended signal processing pipeline

**Fig. 5:**
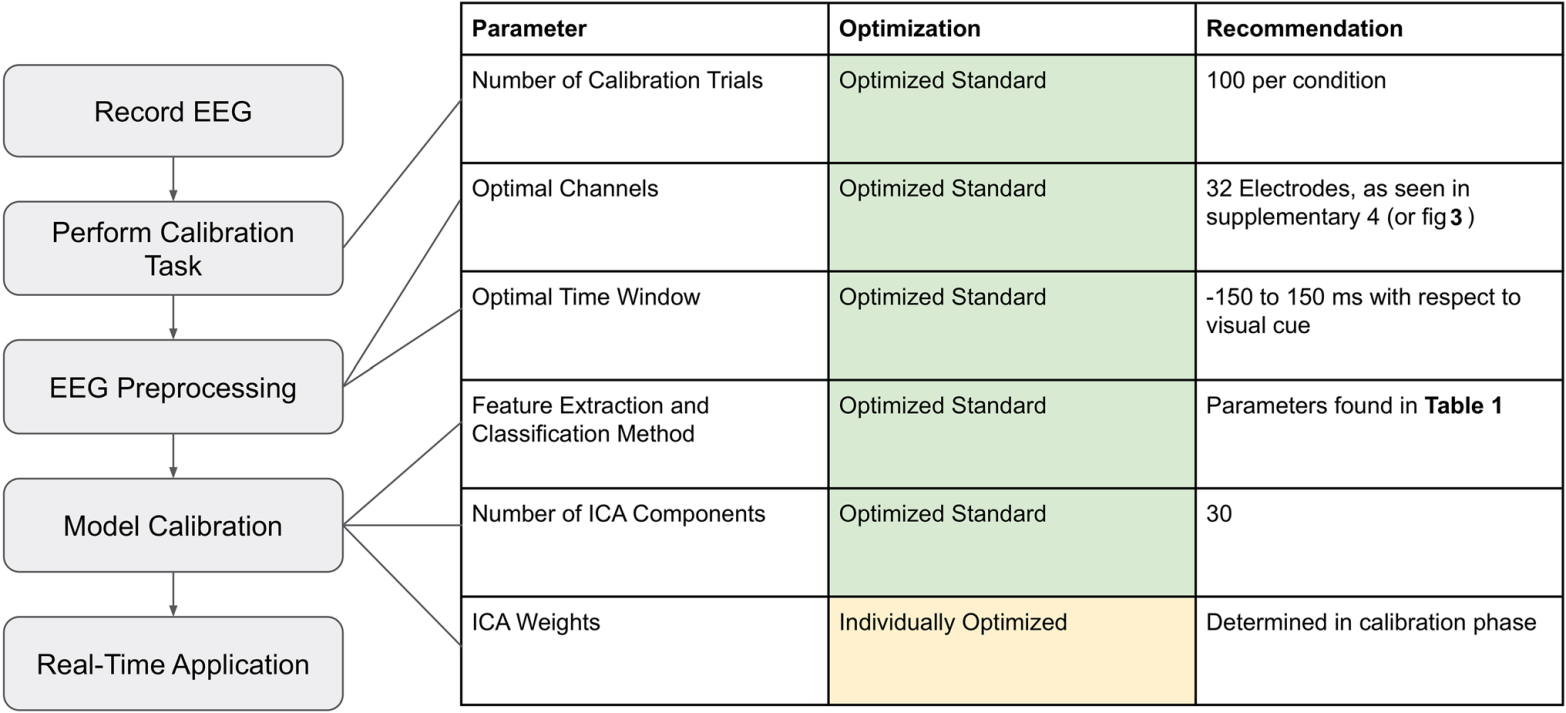
Model steps and recommended parameters. Visualization of pipeline steps, with lines extending to relevant parameters for each step, along with how they are optimized, and our recommendations

The pipeline resulted in effective predictive models for each participant using the following parameters for the classifier: the recommended 32-channels referenced to CPz, a fixed time window of −150 to 150 ms relative to visual stimulus onset, 30 independent components, the participant’s individual ICA weights, and using 100 trials from each condition for calibration. Lambda was determined by the intrinsic “optimize hyperparameters” setting from *fitclinear*. The resulting pipeline is summarized in Fig. 5. Running the classifier on the 20% of each participant’s never-before-seen data, the accuracy was between 69.6% and 97.1%, with an average of 83.3%, comparable to the average of 85.96% obtained when using all available data (all channels, all trials, over the same time window) with the training parameters. The same analysis pipeline when applied to the real-time simulation produced a state-of-the-art 77.1% accuracy across the group when the sliding window was −150 to 150 ms (or 73.3% when testing specifically in the period − 150–0 ms prior to the visual stimulus), however, with a wide variance between participants from 50.9% to 96.5% (Fig. 4c).

## Neurophysiological plausibility

**Fig. 6:**
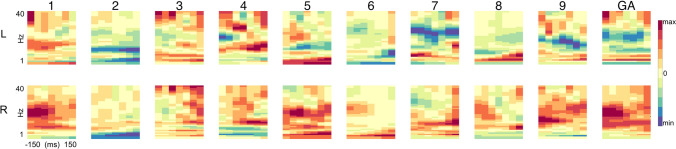
Time frequency analysis (**top row**). Time–frequency (TF) spectrograms for areas-of-interest within the left hemisphere. Each column is one participant, with the tenth column representing the grand average of all participants (GA). The spectrograms were obtained using Morlet wavelets averaged over the areas of C1, C3, CCP1h, FC1, FCC1h, FCC3h. Frequencies range from 2 to 40 Hz across the interval −150 ms to 150 ms. The spectrograms show the normalized difference in spectral power between the selection of right vs. left hand trials (right – left) / (right + left). The color bar represents the max and min values for each subject. (**bottom row)** TF in the right hemisphere averaged over the areas of C2, C4, CCP2h, FC2, FCC2h, FCC4h. The color bar units are normalized to the individual minimum and maximum

Time–frequency analysis was calculated for each participant and for each hemisphere over the average of the area related to the most informative channels and ICA topographies. For the left hemisphere, this involved the electrodes C1, C3, CCP1h, FC1, FCC1h, and FCC3, and for the right hemisphere C2, C4, CCP2h, FC2, FCC2h, and FCC4h. The spectrograms show the normalized difference in spectral power between the selection of right-hand vs. left-hand trials (right − left) / (right + left) for both hemispheres (Fig. 6). This indicates that in those trials, where the right hand is selected, power in the 10–25 Hz band is decreased in the left hemisphere and is increased in the right hemisphere, and vice versa when the left hand is selected, which is consistent with literature concerning pre-movement motor cortical activity (Jasper and Penfield 1949; Pfurtscheller et al. 1998; Tzagarakis et al. 2010).

**Fig. 7:**
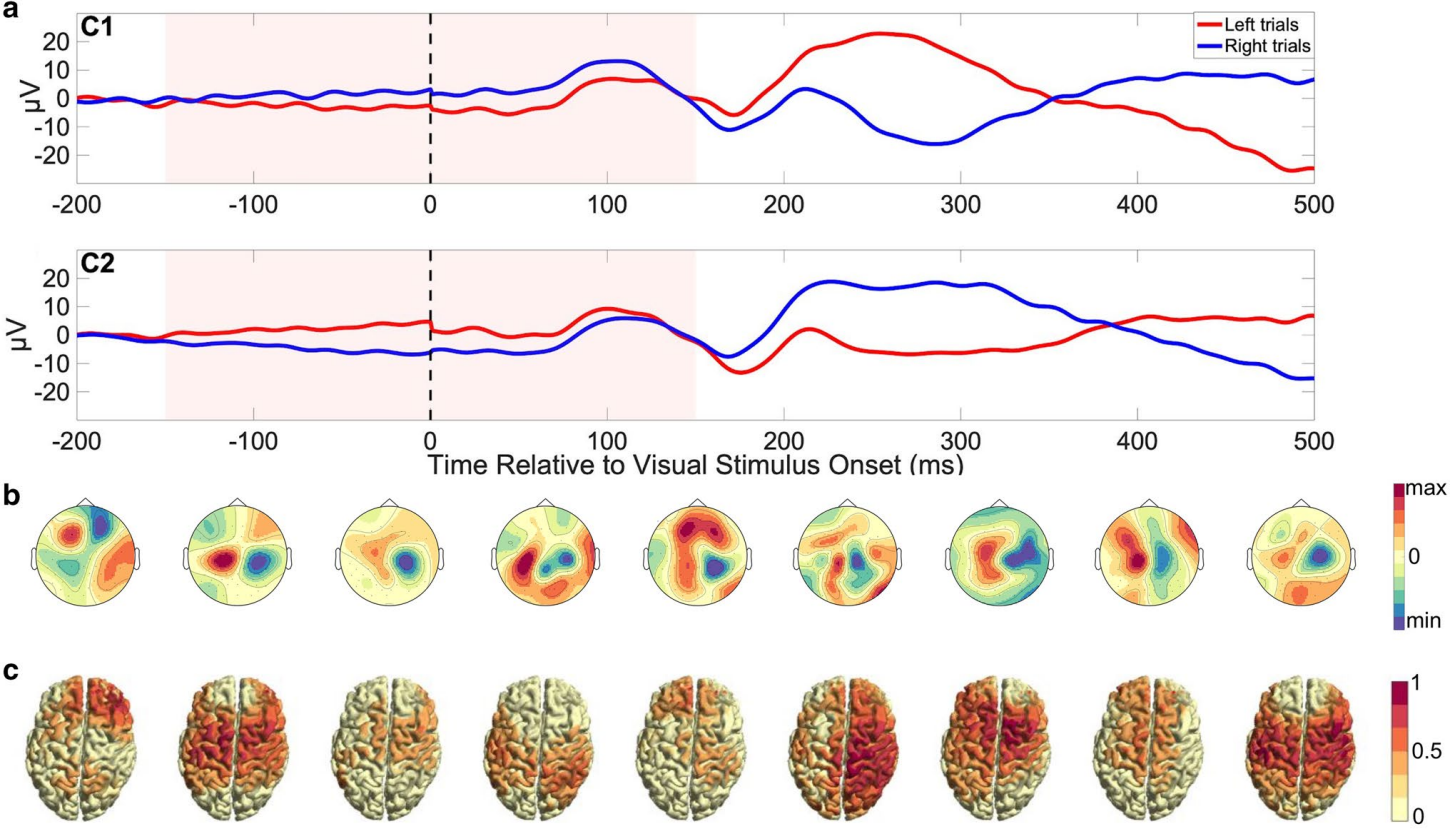
Neurophysiology. **a** The average potential of EEG sensors C1 (left hemisphere) and C2 (right hemisphere) with respect to the reference electrode CPz is shown across all 9 participants, time-locked to the onset of the visual stimulus, for trials where the left hand (red) vs. right hand (blue) was selected. The period used for pipeline classification (−150 to 150 ms) is shaded light red. Median movement onset occurred after 300 ms. **b** The topographies depict each participant’s brain activity pattern most predictive of right- versus left-hand movements within the time window −150 to 150 ms. Topography units are arbitrary and the color scales set to the individual [min max] range. **c** Source localization through Minimum Norm Estimation of classifier-relevant brain activity for each participant, plotted at sensor level. Source power has been normalized to the individual maximum of each participant. With the exception of participant 1, all participants show relevant activity within the sensorimotor network

In order to confirm that the EEG signals derived for classification of movement intention using this pipeline corresponded to neurophysiological relevant brain processes, as opposed to overfitting to artifacts, we calculated the ERP and performed a time–frequency analysis of the most important EEG channels as well as an analysis of the ICA topographies and anatomical source localization. The ERP analysis (Fig. 7a) showed a negative deviation on the hemisphere opposite the side of the hand used in the period 200–350 ms (consistent with studies investigating the lateralized readiness potential (see: Shibasaki and Hallett 2006)). However, note that for the purpose of this study, an earlier time window around the time of the onset of the stimulus was analyzed (before the stimulus can affect the ongoing signal). In our analysis, during this window, a small deviation in the opposite direction before the visual stimulus can be seen. Similar slow fluctuations have previously been shown to modulate motor behavior (Birbaumer et al. 1990), conceptualized in the “slow cortical potentials sampling hypothesis” (Schmidt et al. 2016). Note that in contrast to studies investigating EEG correlates of (self-paced or externally paced) movement, the task used in this study targets spontaneous fluctuations of brain state before motor preparation starts.

The individual topographies of the brain activity most predictive of right- versus left-hand movements are shown in Fig. 7b for each participant, indicating a consistent pattern with a positive deflection in the left hemisphere contrasted with negative deflection localized in the right hemisphere. Reversing the signs of these topographies would represent the pattern predicting left-hand movement (data not shown). For this analysis, we used all available trials and channels, as well as the participants’ individual optimal time windows as defined previously. Localizing the sensor activity to a standard source anatomy as shown in Fig. 7c shows a strong pattern of predictive activity across participants from the sensorimotor area, with exception of participant 1, who also showed amplitude maxima in the frontal channels at sensor level.

As can be seen in our analysis regarding the ranking of relevant channels (Fig. 3c), ICA topographies (Fig. 7b), and source localization (Fig. 7c), the classifier utilizes brain activity originating from motor regions, consistent with the existing literature: MRCPs are typically expressed primarily through the electrodes overlying sensorimotor gyrus (C3, C1, Cz, C2, C4) (Eimer 1998; Schultze-Kraft et al. 2016; Shibasaki and Hallett 2006), but there is also evidence for a role of medial frontocentral cortex as a movement generator (Toma et al. 2002) and the dorsal pre-motor cortex (Lu et al. 2012; Solopchuk et al. 2016) in cued movement preparation.

## Discussion

### Therapeutic relevance

**Fig. 8:**
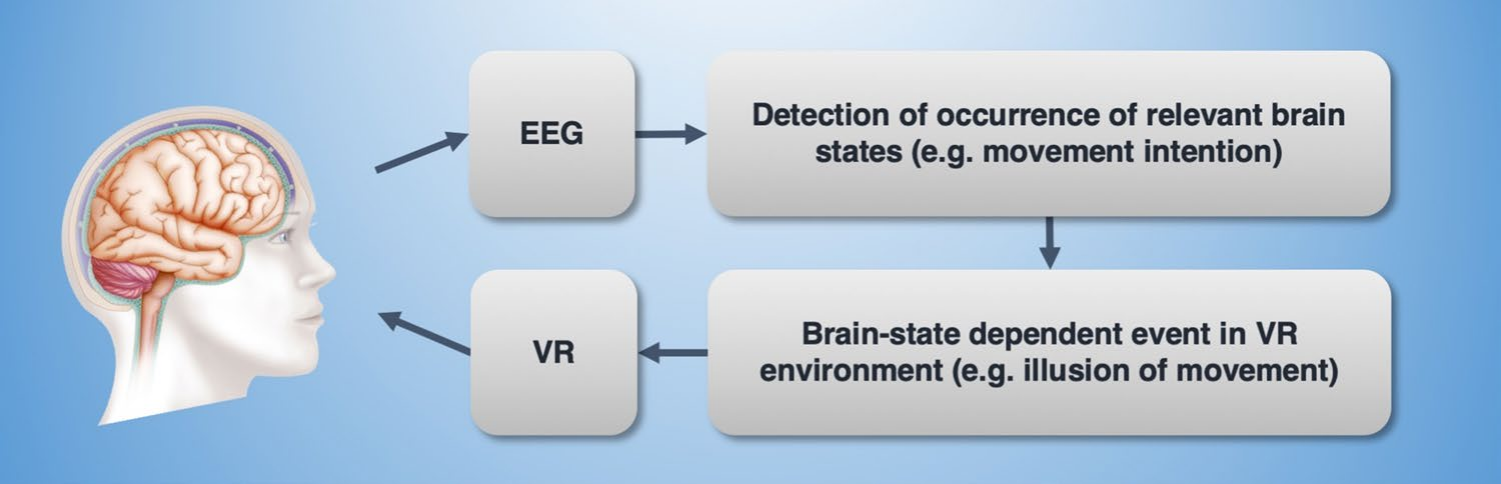
Closed-loop EEG-VR. In a future therapeutic application, where the VR rehabilitation task is controlled by the patient’s own EEG signal, information flows from the EEG recording to a classification system to detect relevant brain states (which is the focus of this study). When a specific target brain state (e.g., related to motor planning) is detected, this information is relayed to the VR controller in order to adapt the environment and optimize the therapy paradigm (e.g., through creating an illusion of movement, as in mirror therapy). The visual stimulation presented through the VR system is perceived by the patient and thereby affects the corresponding EEG signal, thus creating a fully closed-loop system

**Fig. 9:**
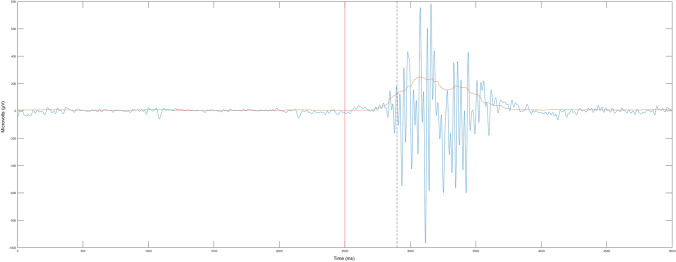
Example of classifying movement onset. EMG trace (light blue) with movement onset detection, enveloped. The vertical red line at 2500 represents the onset of the visual stimulus in the epoch. Movement initiation in this single trial is well over 250 ms after the visual stimulus, as can be seen as the dotted black line

**Fig. 10:**
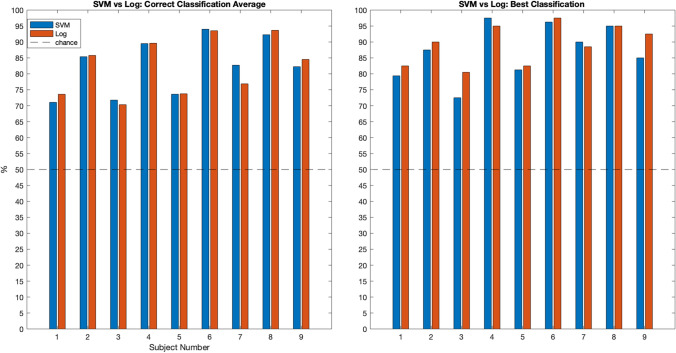
ICA combined with SVM and logistic classifier. To determine which classifier approach works best in combination with ICA, we ran 50 iterations of fivefold cross-validation using the training data. Here, though minimally different, the logistic classifier produces higher average accuracies in 6 of 9 participants, and best accuracies in 7 of 9 participants

To realize the full potential of personalized EEG-VR-based neurorehabilitation, a closed-loop therapy system must “read out” individual evolving brain states (see Fig. 8) in real time to make the same determination that the brain makes when generating a motor action: *What* is the motor intention (appropriate VR-based stimulus, e.g., the illusion of a specific motor action of the paretic hand), as well as *when* to make the movement (synchronized with the patient’s volition) and *whether* to make the movement (requiring a certain threshold)*.* To achieve this, a continuous “decoding” of ongoing EEG activity is required that detects relevant brain states with sufficient accuracy. We have in this study explored different features, feature extraction methods, and classifier models, resulting in a pre-configured EEG decoding pipeline. The pipeline enables both *post hoc* testing of whether a given window of EEG data is predictive of some aspect of a motor behavior, and can also serve as a basis for a real-time implementation.

Since the processing pipeline developed in this study is calibrated using data from healthy study participants, instead of looking for optimal brain states that bias performance (i.e., also including any artifacts), we sought out neurologically relevant brain states that bias laterality. However, any investigation of ongoing fluctuating brain states is complicated by environmental interaction affecting brain dynamics. The specific parameters of the task play a critical role determining the resulting brain states. In choosing a very simple task (“freely reach for the objects that appear” – without any specific instructions regarding hand use), we mimic a natural situation that is regularly impaired by stroke, and therefore directly relevant from a real-world patient-centric point of view (Wright et al. 2011), but we also lose the ability to differentiate different stages of neural processing that is afforded by the use of warning cues.

With respect to classifier performance, real-time single-trial classification has previously been achieved with 65% accuracy in a similar task (Bai et al. 2011; Schultze-Kraft et al. 2017). Whether the 77.1% accuracy achieved in our real-time simulation can in fact be achieved in a “real” real-time situation is yet to be determined, we nevertheless see this as encouraging evidence that this approach may facilitate further development of real-time therapeutic systems designed for the clinical setting.

### Limitations

**Fig. 11:**
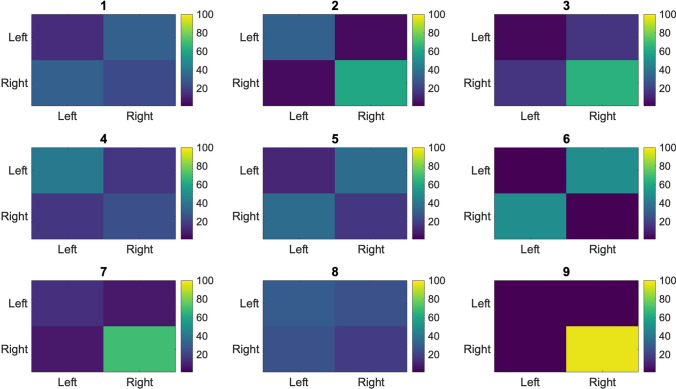
Hand-selection bias. To get a more general idea of pattern tendencies in the participants, each choice and the following choice were tracked, and plotted in four categories (Left-Left, Right-Right, Left–Right, and Right-Left). For example, “Right-Left” would represent a right choice that was preceded by a left choice. The color bars indicate frequency (in %)

An important limitation of this approach is the variability in achievable classification accuracy between participants. One factor influencing the classifier is the quality of the EEG data, but this will also be an issue in a realistic clinical setting, especially when the EEG is affected by movement (this issue is currently being investigated in a follow-up study). A further limitation with respect to the interpretability of the targeted EEG signal as corresponding to an “ongoing fluctuating state” is the use of a repetitive task in which participants could become accustomed to the timing of the stimulus onset. We attempted to reduce anticipation effects by using a jitter in that the latency to the next stimulus varied between 2.5 and 3.5 s after returning the hands to the home position. Participants were not instructed with regard to using the left or right hand to avoid any cognitively generated bias; however, this led to some participants using predominantly one hand or alternating hands (see Appendix 1, Fig. 11 for a movement matrix), which both reduced the randomness of the pre-stimulus brain state we intended to identify, and also the number of usable trials (which were balanced between classes to reduce bias). A task where explicit randomization of left- versus right-hand use is employed by instructing participants not to be predictable may be effective in producing more balanced behaviors (see “*Matching Pennies*” in Schultze-Kraft et al. (2017)).

Concerning the parameters explored in determining the classification pipeline, we did not, and could not, exhaustively test all possible combinations and approaches. We chose to test an arbitrary set of choices with respect to features, feature extraction, and classification approaches, based on availability of toolboxes and popularity in the literature, as well making some pragmatic choices with respect to the goal of the study and the task design. Finally, whereas we were able to propose standard settings for many parameters, and the model creation portion of the pipeline takes only a few minutes, we also recommend a 100-trial acquisition per condition calibration step. For our task, this takes approximately 15 min. However, given the similarity in the ICA topographies and the consistent activity in the time–frequency analysis, we expect that by using suitable priors, a sufficient calibration should be possible with fewer trials, leading to a significant acceleration of the calibration, as was achieved previously by Jayaram et al. (2016).

### Outlook

Whereas “detectability” is a necessary requirement for a given brain state to serve as a therapeutic target in a closed-loop EEG-VR paradigm, this alone is not sufficient. We will also need to demonstrate “therapeutic relevance”, in that synchronizing a virtual neurorehabilitation task with a given brain state improves the long-term outcome for the patient. One characteristic of potentially relevant brain states is that they affect motor performance, making it more or less likely that an impaired motor function is executed successfully. This approach is based on the hypothesis that if a VR-based rehabilitation task is repeatedly executed during periods where it is more likely to be performed well, it improves the effectiveness of the therapy.

We would also like to point out that even a suboptimal classification accuracy, constrained by what is achievable in a clinical neurorehabilitation setting, may already lead to a significant patient benefit: This work is based on the hypothesis that a given VR-based rehabilitative motor task is therapeutically most effective when it is triggered during a specific brain state, which can be estimated through EEG. If a spontaneously occurring target brain state (e.g., synchronicity between the left and right motor cortex) is matched by chance (without using the EEG signal to control the VR task) only rarely (say, 10% of the trials), achieving an accurate match even 50% of the time would achieve a fivefold increase in correctly matched training events. Clinical trials are needed to determine how such an approach translates to clinical outcome.

In conclusion, we hope that this study will enable experimenters, clinicians, and therapists to quickly prepare VR-based therapy that is linked to ongoing fluctuating brain states; and to venture into a scenario where the patient’s brain creates the virtual environment that in turn supports brain network reorganization in a true “closed-loop” fashion. This allows for applications beyond mirror therapy, such as visually experiencing the illusion of successful movements triggered by imagination and intention, controlling the type and timing of VR-based therapy tasks, and presenting stimuli at the moments most conducive to executing a successful movement, as determined by the individual’s own brain state.

## Figures and Tables

**Table:**
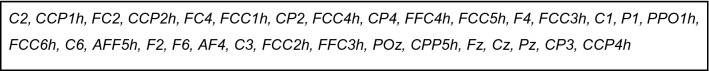
Supplementary Item 3: Recommended 32-channel electrode set, referenced to CPz

**Table:**
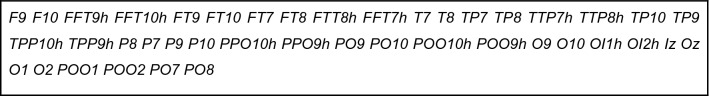
Supplementary Item 4: List of removed peripheral electrodes

**Table:**
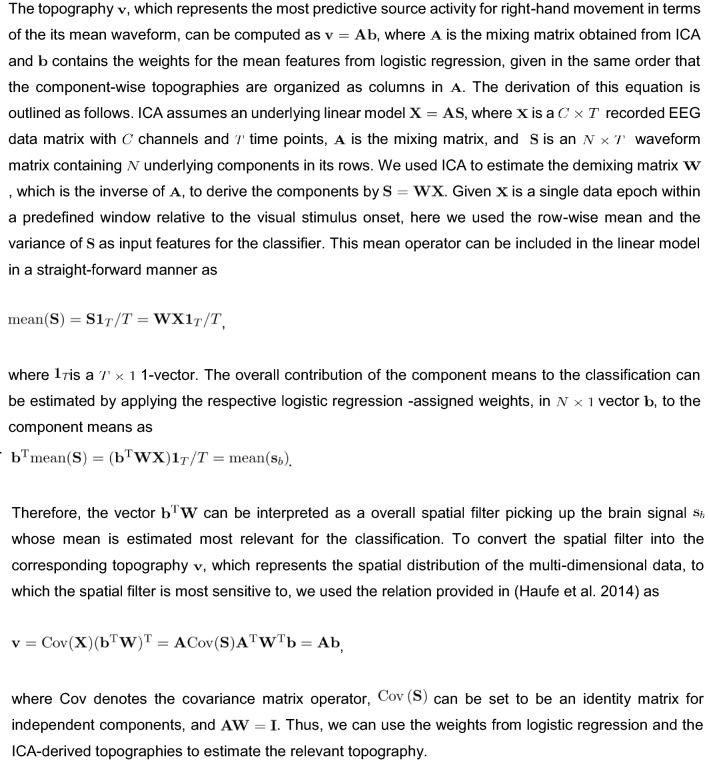
Supplementary Item 6: Computing the individual most predictive topographies

## Data Availability

All toolboxes are openly available, MATLAB requires a software license, and the Leap Motion hand tracking system can be purchased commercially. Code is available for download at following location: https://github.com/bnplab/rightleft.

## Appendix 1: Additional figures

See Figs. 9, 10, 11.

## Appendix 2: Definitions, lists, and formulas

**Table:** Supplementary Item 1: Technical Concepts and Definitions (quoting from references specified)

**Principle component analysis (PCA)**: an orthogonal linear transformation that transforms the data to a new coordinate system such that the greatest variance by some scalar projection of the data comes to lie on the first coordinate (called the first principal component), the second greatest variance on the second coordinate, and so on (Jolliffe 2002)
**Independent component analysis (ICA)**: a probabilistic method for learning a linear transform of a random vector. The goal is to find components that are maximally independent and non-Gaussian (non-normal). Its fundamental difference to classical multivariate statistical methods is in the assumption of non-Gaussianity, which enables the identification of original, underlying components, in contrast to classical methods (Hyvärinen 2013)
**Support vector machine (SVM)**: supervised learning models with associated learning algorithms that analyze data for classification and regression analysis (Cortes and Vapnik 1995). They construct a hyperplane or set of hyperplanes in a high- or infinite-dimensional space, which can be used for classification, regression, or other tasks like outliers detection. Intuitively, a good separation is achieved by the hyperplane that has the largest distance to the nearest training-data point of any class (so-called functional margin), since in general the larger the margin, the lower the generalization error of the classifier (Hastie et al. 2009)
**EEG source localization**: The human brain continuously generates electromagnetic signals. The localization of the active brain areas which are responsible for those signals is termed as brain source localization. This process of source estimation with the help of EEG is known as the EEG source localization problem, a problem which has both a forward problem (estimating the potentials at the electrodes on the scalp, given some source distribution inside the head) and an inverse problem (estimate the distribution of source(s) from an EEG recording). In the current study, we used minimum norm estimation (MNE)
MNE is routinely employed for EEG evoked responses, revealing wide-spread activation in the different cortical areas. It is a distributed inverse solution that discretizes the source space into mesh points on the cortical surface using an elevated number of equivalent current dipoles. It estimates cortical activity power on the source locations simultaneously, choosing the distribution that exerts the minimum overall energy to represent sensor data consistent with the recorded ones
**Time–frequency analysis (with Morlet wavelets)**: Wavelet Transform, a typical time–frequency domain method, can extract and represent properties from transient biological signals. Specifically, through wavelet decomposition of the EEG records, transient features can be accurately captured and localized in both time and frequency context. Thus wavelet transform is like a mathematical microscope that can analyze different scales of neural rhythms and investigate small-scale oscillations of the brain signals while ignoring the contribution of other scales (Adeli et al. 2003; Hazarika et al. 1997)
**Event-related potential (ERP)**: is the measured brain response that is the direct result of a specific sensory, cognitive, or motor event. More formally, it is any stereotyped electrophysiological response to a stimulus (Luck 2014). Typically, ERPs are scalp recorded voltage fluctuations that are time-locked to an event. The ERPs reflect stages of information processing in the sensory-related hierarchical neuronal networks, in the networks of cognitive control as well as in the memory and affective systems of the brain. The ERP amplitude is usually smaller than the amplitude of background EEG so that the reliable ERP is obtained by averaging EEG fragments in multiple trials. Each ERP represents a sum of potentials generated in widely distributed cortical sources. The functionally different sources of ERPs are called components and are associated with distinct hypothetical psychological operations. One way of separating the components is to obtain a difference between the two ERPs performed at conditions which differ in only one respect: presence or absence of a hypothetical operation (Kropotov 2016)
**Event-related (De)synchronization (ERD/S)**: ERD designates a short-lasting and localized amplitude attenuation of rhythms within the alpha band; event-related synchronization (ERS) describes a short-lasting amplitude enhancement. The topographical pattern of the ERD may reflect activation or excitation of cortical areas. Localized patterns or ERS probably represent inhibition of cortical areas (Pfurtscheller 1991)
**Contingent negative variation (CNV)**: the reaction time between a warning and a *go* signal as measured by EEG. The CNV was one of the first event-related potential (ERP) components to be described. In a chronometric paradigm, the first stimulus is called the warning stimulus and the second stimulus, often one that directs the subject to make a behavioral response, is called the imperative stimulus. The foreperiod is the time between the warning and imperative stimuli. The time between the imperative stimulus and the behavioral response is called the reaction time. The CNV, then, is seen in the foreperiod, between the warning and imperative stimulus (Walter et al. 1964)
**Bereitschaftspotential (BP)**: a measure of activity in the motor cortex and supplementary motor area of the brain leading up to voluntary muscle movement. The BP is a manifestation of cortical contribution to the pre-motor planning of volitional movement. Note that the BP has two components, the early one (BP1) lasting from about − 1.2 to − 0.5; the late component (BP2) from − 0.5 to shortly before 0 s (Deecke et al. 1976; Kornhuber and Deecke 1965)
**Lateralized Readiness Potential (LRP)**: an event-related brain potential, or increase in electrical activity at the surface of the brain, that is thought to reflect the preparation of motor activity on a certain side of the body; in other words, it is a spike in the electrical activity of the brain that happens when a person gets ready to move one arm, leg, or foot. It is a special form of the BP (Vaughan Jr et al. 1968)

**Table:** Supplementary Item 2: Machine learning background (quoting from references specified)

Machine learning (ML) is the study of computer algorithms that automatically improve through experience (Mitchell 1997). ML algorithms build a model based on sample data, known as "training data", in order to make predictions or decisions without being explicitly programmed to do so (Koza et al. 1996). In supervised learning, the approach taken by the work at hand, the computer is presented with example inputs and their corresponding outputs, the goal is to then learn a general rule to map inputs to outputs, a so-called input–output pair (Russell and Norvig 2002). Through iterative optimization of an objective function, supervised learning algorithms learn a function that can be used to predict the output associated with new inputs (Mohri et al. 2012). An optimal function will allow the algorithm to correctly determine the output for inputs that were not a part of the training data. An algorithm that improves the accuracy of its outputs or predictions over time is said to have learned to perform that task (Mitchell 1997). Types of supervised learning algorithms include active learning, classification and regression. The algorithm that then maps these input–output pairs would be known as the “classifier”. However, prior to this mapping, the data must be prepared in a way that is optimized. Typically, choices must be made with respect to dimensionality reduction (i.e., feature selection and feature extraction (Pudil and Novovičová 1998)), and algorithm used for the classification
We chose, for instance, to use a linear classifier. In general, a linear classifier achieves input–output mapping by making a classification decision based on the value of a linear combination of the characteristics. An object's characteristics are also known as feature values and are typically presented to the machine in a vector called a feature vector. Such classifiers work well for practical problems such as document classification, and more generally for problems with many variables (features), reaching accuracy levels comparable to nonlinear classifiers while taking less time to train and use (Yuan et al. 2012). Apart from that, we used PCA and ICA for dimensionality reduction
Figure taken from (Cozza et al. 2020) with permission

**Table:** Supplementary Item 5: Enveloped EMG analysis

*Step 1: Filter Design*
Filter = designfilt('highpassfir', 'FilterOrder', 100, 'CutoffFrequency', 10, 'SampleRate', 1000)
*Step 2: Envelope*
e = envelope(data_filtered, 100, 'rms');
*Step 3: Threshold*
movementOnset = find(e > = 0.5 * max(e(MOVWINDOW)), 1); % at some percentage of max envelope
movementOnset = find(e > = 0.5 * max(e(MOVWINDOW)), 1); % at some percentage of max envelope

**Table:** Supplementary Item 7: Fieldtrip electrode layout and head model

https://github.com/fieldtrip/fieldtrip/blob/master/template/electrode/standard_1005.elc
https://github.com/fieldtrip/fieldtrip/blob/master/template/headmodel/standard_bem.mat
